# Rosai-Dorfman-Destombes (RDD) disease presenting as palindromic rheumatism

**DOI:** 10.1186/s12880-021-00596-2

**Published:** 2021-04-15

**Authors:** Amin Haghighat Jahromi, Aaron M. Goodman, Carl K. Hoh

**Affiliations:** 1 Department of Radiology, University of California, UCSD Medical Center, 200 W Arbor Drive, San Diego, CA 92103-2686 USA; 2 Department of Medicine, Division of Blood and Marrow Transplantation, Moores Cancer Center, University of California, San Diego, CA USA

**Keywords:** Sinus histiocytosis with massive lymphadenopathy, Rosai-Dorfman-Destombes disease, Histiocytosis, Palindromic rheumatism, ^18^F-FDG PET/CT

## Abstract

**Background:**

Rosai-Dorfman-Destombes (RDD) disease, is a rare proliferative and inflammatory disorder of non-Langerhans cell histiocytes.

**Case presentation:**

We report a 35-year-old woman, who originally presented with recurrent episodes of lower extremity joint/bone pain and chronic nasal stuffiness and congestion. Her worsening nasal congestion was due to an obstructing nasal cavity lesion which was subsequently biopsied. Pathology was consistent with RDD. ^18^F-FDG PET images demonstrated intense uptake in the paranasal sinuses and a large pelvic lymph node. Focal osseous lesions with intense ^18^F-FDG uptake were also observed in the lower extremity, corresponding to areas of peri-articular pain. Rheumatologic work-up was consistent with palindromic rheumatism. She was diagnosed with immune-related disseminated RDD, presenting as palindromic rheumatism.

**Conclusions:**

This is the first case of RDD presenting as palindromic rheumatism. RDD should be considered as a possible but rare diagnosis in young patients with sinus-related symptoms and lymphadenopathy. The disease can on rare occasions be disseminated and can also present as immune-related RDD, such as in this patient.

## Background

Rosai-Dorfman-Destombes (RDD) disease also known as sinus histiocytosis with massive lymphadenopathy (SHML), is a rare heterogeneous disease of children and young adults, that can occur in association with autoimmune disorders, hereditary diseases, and malignancies [1, 2]. The prevalence of RDD is 1:200,000, with an average age of onset of 20.6 years, and occurs more commonly in African patients with a slight male predominance (male to female ratio of 1.4) [1, 3, 4]. The disease is a non-Langerhans cell histiocytosis characterized by accumulation of activated histiocytes, presenting as nodal, or extranodal disease within affected tissues [1]. We describe a young patient with a constellation of non-specific symptoms and findings including stuffy nose, pelvic lymphadenopathy and lower extremity joint pain, diagnosed with palindromic rheumatism, a novel presentation of RDD. This is the first report of RDD presenting as palindromic rheumatism, to the best of our knowledge.

## Case presentation

A 35-year-old woman had originally presented with recurring attacks of lower extremity joint/bone pain and chronic nasal stuffiness and congestion. With a diagnosis of palindromic rheumatism and chronic rhinosinusitis for 9 years, she had been treated with intermittent corticosteroids, typically methylprednisolone 40 mg for 5 days duration. Upon gradual progression of her nasal congestion, she was found to have obstructing nasal cavity lesion on physical examination which was subsequently biopsied. Pathology of the sample was consistent with RDD, demonstrating proliferation of macrophages devoid of cytologic atypia in which emperipolesis was easily identifiable. The macrophages had open chromatin, distinct nucleoli and eosinophilic or foamy cytoplasm. Macrophages were S100^+^, CD1a^−^, and langerin negative. Plasma cells within the expanded medullary cords were IgG-positive. IgG4 positive plasma cells accounted for 20% of the plasma cells.

**Fig. 1:**
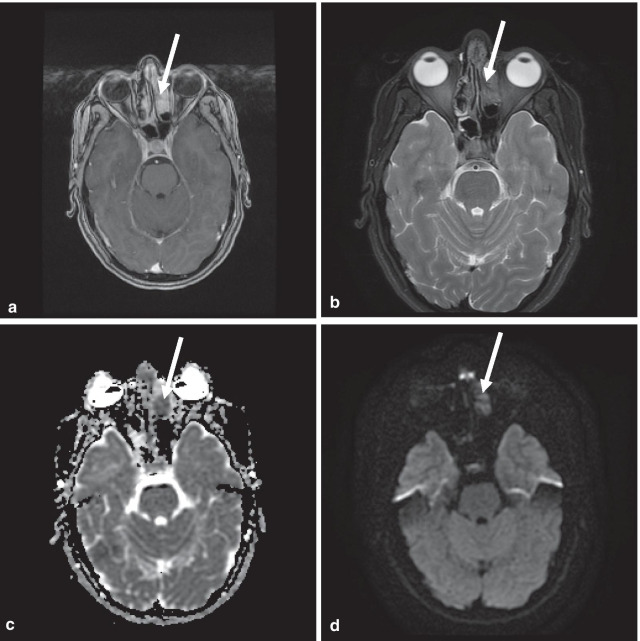
Contrast-enhanced maxillofacial MRI demonstrates left ethmoid sinus lesion. **a** Enhancing infiltrative lesion in the left anterior ethmoidal sinus in the Gadolinium-enhanced sequence (White arrow). **b** This lesion is hypointense in the T2-weighted sequence. **c**, **d** The lesion is hypointense in the apparent diffusion coefficient (ADC) sequence and mildly hyperintense in the diffusion-weighted imaging (DWI) sequence, likely due to hypercellularity

**Fig. 2:**
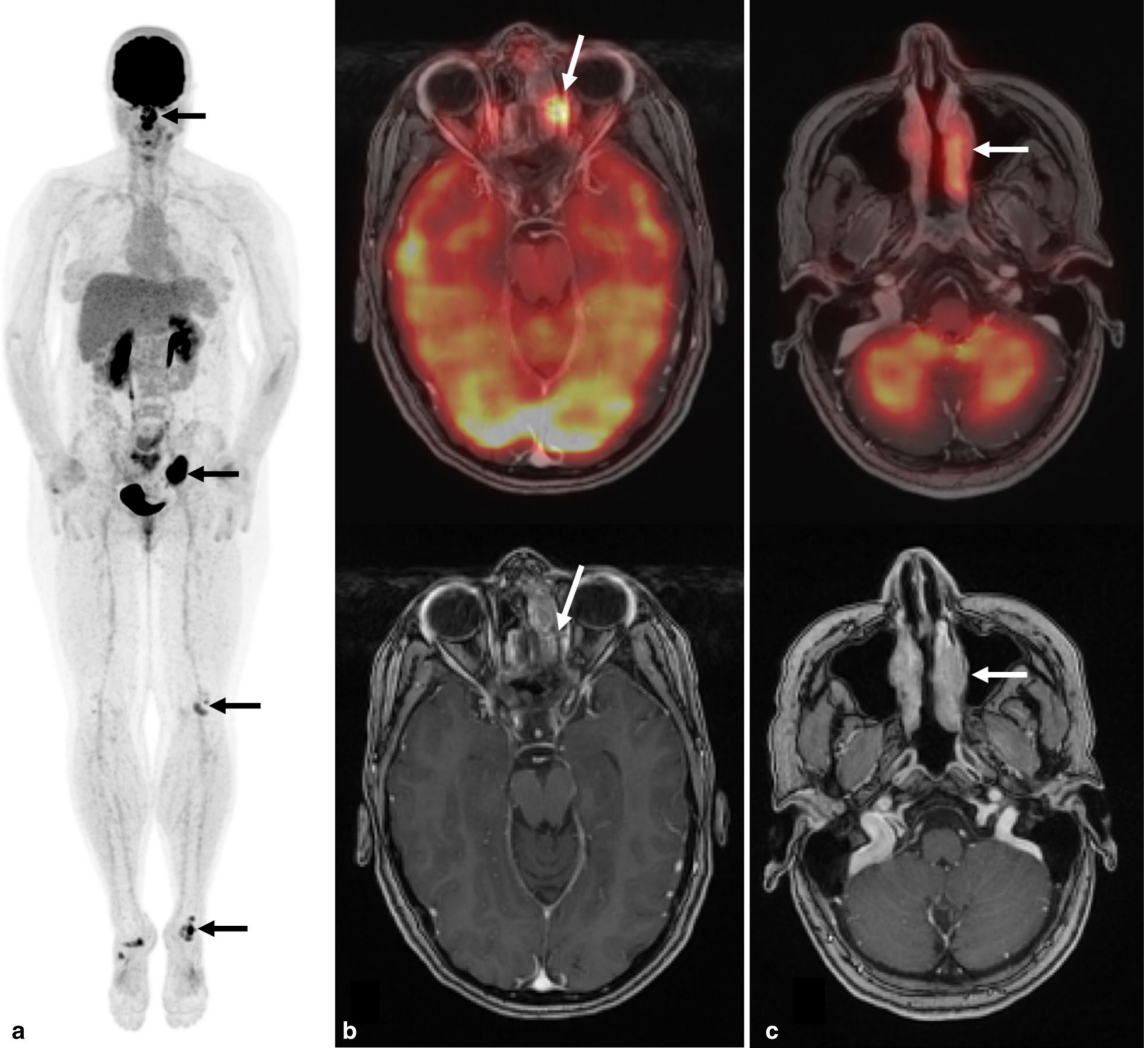
Whole-body ^18^F-FDG PET co-registered with contrast-enhanced maxillofacial MRI demonstrates sinus lesions. **a** Whole-body FDG PET maximum intensity projection image demonstrates intense uptake in the nasal cavity, paranasal sinuses, left pelvis, left knee and left ankle (black arrows). Nonspecific mild uptake in the right ankle, and left side of the neck are also seen. **b** Mildly enhancing infiltrative lesion in the left anterior ethmoidal sinus in the Gadolinium-enhanced maxillofacial MRI, demonstrates intense FDG uptake in the fused PET/MRI axial image (SUV_max_ 10.6, white arrow). **c** Enhancing infiltrative mass in the left middle nasal turbinate, in the Gadolinium-enhanced maxillofacial MRI, demonstrates intense FDG uptake in the fused PET/MRI axial image (SUV_max_ 7.7, white arrow)

**Fig. 3:**
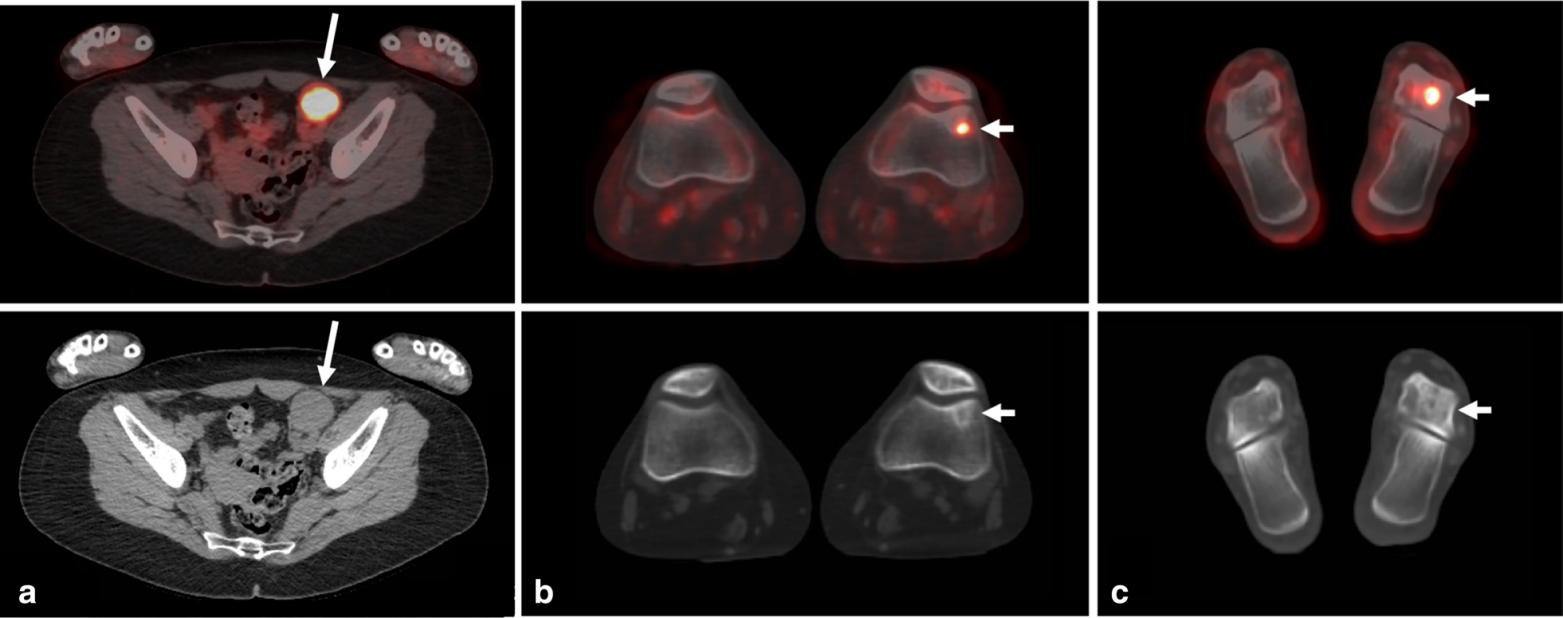
Whole-body ^18^F-FDG PET/CT demonstrates pelvic lymphadenopathy and osseous lesions. **a** Intense uptake in a 3.8 × 3.6 cm left pelvic side wall lymph node on the fused PET/CT axial image (SUV_max_ 9.8, white arrow). **b** Moderate FDG uptake in the left lateral femoral condyle (SUV_max_ 3.1, white arrow), on the fused PET/CT axial image, correlating with the mixed lytic/sclerotic subchondral lesion in the CT. **c** Intense FDG uptake in the left talus on the fused PET/CT axial image (SUV_max_ 7.7, white arrow) correlating with mixed lytic/sclerotic subchondral lesion on the CT

Imaging evaluation consisted of gadolinium-enhanced maxillofacial MRI (Fig. 1), to assess the locoregional extent of the disease, and also subsequent whole body ^18^F-FDG PET/CT, for staging of the disease. The MRI showed a non-specific T2-hypointense enhancing infiltrative lesion in the left anterior ethmoid sinus and middle nasal turbinate which was FDG-avid on PET/CT (Figs. 1 and 2b, c). ^18^F-PET/CT also showed a large FDG-avid left pelvic lymph node (Fig. 2a), and focal FDG-avid mixed lytic/sclerotic subchondral lesions in the left lateral femoral condyle and left talus (Fig. 3b, c).

Her rheumatologic work-up was positive for Anti-Nuclear Antibody (ANA), and negative for other autoimmune/inflammatory antibodies including RF, anti-CCP, anti-smith, anti-ds-DNA, TPO, C3, C4, anti-SSA and anti-SSB. Diagnosis of palindromic rheumatism was confirmed based on her work-up, and history of recurrent attacks of arthritis/peri-arthritis without joint space loss or erosion. RDD can behave as a malignant disease with somatic alterations [1, 4], thus she underwent peripheral blood next-generation sequencing which did not detect any genomic alterations, consistent with benign RDD.

She underwent maxillary antrostomy, total ethmoidectomy, and frontal sinus exploration to resect the infiltrative nasal mass. Sinonasal surgery helped her nasal obstruction. Currently three years after the surgery, her sinonasal symptoms are under control, by regular saline irrigation and Budesonide rinse, twice a day. She received Methotrexate, 25 mg weekly, and prednisolone, approximately twice a year, for palindromic rheumatism flares. For staging evaluation of RDD she undergoes clinical follow-up including ^18^F-FDG PET/CT, semiannually. Although her palindromic rheumatism flares and sinonasal symptoms have been under control for three years, her recent ^18^F-FDG PET/CT demonstrated progressive disease with new hypermetabolic pericardial involvement, in keeping with her new symptom of pleuritic chest pain. Methotrexate was switched to cladribine 5 mg/m^2^ on days 1–5 every 28 days, per expert guideline, considering limited data to guide therapy in this rare disease [1]. She is currently on the third cycle of cladribine. Her most recent ^18^F-FDG PET/CT demonstrated favorable treatment response with decreased sinonasal disease burden and decreased pericardial effusion size and metabolism.

## Discussion

RDD is caused by proliferation of non-Langerhans histiocytes that are S100^+^, CD68^+^, CD1a^−^ and demonstrate emperipolesis [5]. Commonly, RDD presents as a nodal type, exclusively involving lymph nodes resulting in lymphadenopathy. Extranodal SHML is less common, and manifests as lesions in extranodal organs such as the skin, upper respiratory tract, and bone. Very rarely, SHML is disseminated involving both nodal and extranodal organs, such as this case report. Although usually benign, SHML can behave as a malignant disease with NRAS, KRAS, MAP2K1, and ARAF somatic alterations in as many as 40% of patients [6]. RDD diagnosis warrants careful attention to autoimmune disease because of coexistence of autoimmune disease and SHML in 10% of cases [1]. There are reports of coexistence of SHML with systemic lupus erythematosus, idiopathic juvenile arthritis, autoimmune hemolytic anemia and autoimmune leukoproliferative disease [7–9]. There has also been one case report of RDD associated with rheumatoid arthritis [10]. However, to the best of our knowledge, palindromic rheumatism has not been previously reported as an immune-related RDD. Palindromic rheumatism is characterized by sudden, transient and recurrent flares of peri-articular pain. It is an overlap syndrome, with both autoimmune and autoinflammatory periodic mono or oligo-arthritis and peri-arthritis, clinically and radiologically distinct from rheumatoid arthritis [11]. RDD is a heterogeneous disease, thus depending on the form of the disease i.e.*,* nodal, extranodal and cutaneous, it can have a wide range of differential diagnoses including rhinosinusitis, lymphoma, infectious/disease, metastatic disease, and other histiocytses. This case had been diagnosed with chronic rhinosinusitis and palindromic rheumatism for a long time until the nasal mass was detected on physical exam.

RDD lesions are FDG avid, presumably because of the cellular proliferation and inflammation [12, 13]. Diagnostic and staging evaluation of RDD is done by ^18^F-FDG PET/CT, similar to other histiocytoses such as Erdheim-Chester disease and Langerhans-cell histiocytosis [1, 5]. In children, judicious use of PET/CT is recommended, to minimize radiation exposure and the need for anesthesia. Whole-body PET scan should include distal extremities to evaluate osseous lesions, such as in this case. Whole-body contrast-enhanced MRI is an alternative for staging, especially to evaluate extranodal lesions such as brain, orbit, gastrointestinal or genitourinary lesion which are less favorably evaluated by FDG PET [1, 14].

In conclusion, we report presentation of disseminated RDD with palindromic rheumatism. This is the first report of palindromic rheumatism as an immune-related SHML. Appearance, avidity, and distribution of SHML lesions in ^18^F-FDG PET are similar to lymphoma, metastatic disease, and other inflammatory diseases such as other subtypes of histiocytosis. It is important for nuclear medicine radiologists to be familiar with RDD as a differential diagnosis in young patients with combination of FDG-avid infiltrative lesions in paranasal sinuses and lymphadenopathy, besides the more common infectious, inflammatory, and neoplastic diseases.

## Conclusion

Rarity of Rosai-Dorfman-Destombes (RDD) disease, leads to difficulty in diagnosis and treatment. Most cases are undiagnosed children presenting with many years of sinonasal symptoms, previously attributed to chronic rhinosinusitis. RDD should be in the differential diagnosis of a child or young adult presenting with chronic sinus related symptoms and nasal mass with or without lymphadenopathy. We present palindromic rheumatism as a novel presentation of RDD, indicating immune-related disease. Constellation of hypermetabolic lymph nodes and nasal/sinus lesions in ^18^F-FDG PET in young age should raise the suspicion for RDD, besides the more common infectious, inflammatory, and neoplastic diseases.

## Data Availability

Relevant data are available upon request.

## Abbreviations

SHML: Sinus histiocytosis with massive lymphadenopathy
FDG: Fluorodeoxyglucose
PET: Positron emission tomography
CT: Computed tomography
MRI: Magnetic resonance imaging
SUV: Standardized uptake value
ANA: Anti-Nuclear Antibody

## References

[1] Abla O, Jacobsen E, Picarsic J, Krenova Z, Jaffe R, Emile JF, Durham BH, Braier J, Charlotte F, Donadieu J (2018). Consensus recommendations for the diagnosis and clinical management of Rosai-Dorfman-Destombes disease. Blood.

[2] Menon MP, Evbuomwan MO, Rosai J, Jaffe ES, Pittaluga S (2014). A subset of Rosai-Dorfman disease cases show increased IgG4-positive plasma cells: another red herring or a true association with IgG4-related disease?. Histopathology.

[3] Foucar E, Rosai J, Dorfman R (1990). Sinus histiocytosis with massive lymphadenopathy (Rosai-Dorfman disease): review of the entity. Semin Diagn Pathol.

[4] Bruce-Brand C, Schneider JW, Schubert P (2020). Rosai-Dorfman disease: an overview. J Clin Pathol.

[5] Emile JF, Abla O, Fraitag S, Horne A, Haroche J, Donadieu J, Requena-Caballero L, Jordan MB, Abdel-Wahab O, Allen CE (2016). Revised classification of histiocytoses and neoplasms of the macrophage-dendritic cell lineages. Blood.

[6] Diamond EL, Durham BH, Haroche J, Yao Z, Ma J, Parikh SA, Wang Z, Choi J, Kim E, Cohen-Aubart F (2016). Diverse and targetable kinase alterations drive histiocytic neoplasms. Cancer Discov.

[7] Vaiselbuh SR, Bryceson YT, Allen CE, Whitlock JA, Abla O (2014). Updates on histiocytic disorders. Pediatr Blood Cancer.

[8] Ragotte RJ, Dhanrajani A, Pleydell-Pearce J, Del Bel KL, Tarailo-Graovac M, van Karnebeek C, Terry J, Senger C, McKinnon ML, Seear M (2017). The importance of considering monogenic causes of autoimmunity: A somatic mutation in KRAS causing pediatric Rosai-Dorfman syndrome and systemic lupus erythematosus. Clin Immunol.

[9] Lopetegui-Lia N, Asad SD, Jafri SI, Harrison JS (2019). Autoimmune diseases and Rosai-Dorfman disease coexist more commonly than expected: two case reports. Am J Case Rep.

[10] Gupta S, Finzel KC, Grubber BL (2004). Rosai-Dorfman disease masquerading as chronic ankle arthritis: a case report and review of the literature. Rheumatology (Oxford).

[11] Sanmarti R, Cañete JD, Salvador G (2004). Palindromic rheumatism and other relapsing arthritis. Best Pract Res Clin Rheumatol.

[12] Xue Q, Miao W (2017). Spontaneous recovery of Rosai-Dorfman disease on FDG PET/CT. Clin Nucl Med.

[13] Karunanithi S, Singh H, Sharma P, Naswa N, Kumar R (2014). 18F-FDG PET/CT imaging features of Rosai Dorfman disease: a rare cause of massive generalized lymphadenopathy. Clin Nucl Med.

[14] Sher AC, Orth R, McClain K, Allen C, Hayatghaibi S, Seghers V (2017). PET/MR in the assessment of pediatric histiocytoses: A comparison to PET/CT. Clin Nucl Med.

